# Bridging the gap to effective feedback in residency training: perceptions of trainees and teachers

**DOI:** 10.1186/s12909-018-1333-9

**Published:** 2018-10-03

**Authors:** Brendan M. Carr, Amy O’Neil, Christine Lohse, Stephanie Heller, James E. Colletti

**Affiliations:** 1 Department of Emergency Medicine, Mayo Clinic, 200 First St. SW, Rochester, MN 55904 USA; 2 Department of Emergency Medicine, University of Minnesota Medical Center, Minneapolis, MN USA; 3 Division of Biomedical Statistics and Informatics, Mayo Clinic, Rochester, MN USA; 4 Division of Trauma, Critical Care, and General Surgery, Department of Surgery, Mayo Clinic, Rochester, MN USA

**Keywords:** Feedback, Residency education, Clinical evaluation

## Abstract

**Background:**

Clinical feedback is an important part of residency training, yet literature suggests this complex interaction is not completely understood. In particular, little is known about what resident versus attending physicians expect as feedback. This study investigates this gap in knowledge by examining differences in interactions that residents and attendings view as feedback.

**Methods:**

Surveys containing sample clinical feedback scenarios were distributed to residents and attending physicians in emergency medicine and general surgery at a large academic medical center. Respondents were asked to decide whether useful feedback was provided in each scenario, and responses were compared between the two groups.

Continuous features were summarized with medians, interquartile ranges (IQRs), and ranges; categorical features were summarized with frequency counts and percentages. Comparisons of features between residents and attendings were evaluated using Wilcoxon rank sum, chi-square, and Fisher exact tests. Statistical analyses were performed using version 9.4 of the SAS software package (SAS Institute, Inc.; Cary, NC). All tests were two-sided and *p*-values < 0.05 were considered statistically significant.

**Results:**

Seventy-two individuals responded to the survey out of approximately 110 invitations sent (65%), including 35 (49%) residents and 37 (51%) attendings. Of 35 residents, 31 indicated their level of training, which included 13 (42%) PGY-1, 9 (29%) PGY-2, 6 (19%) PGY-3, and 3 (10%) PGY-4, respectively. Of 37 attendings, 34 indicated the number of years since completion of residency or last fellowship, at a median of 9 years (IQR 4–14; range 1–31). No significant difference was found in residents’ and attendings’ perceptions of what constituted feedback in the sample scenarios.

**Conclusions:**

While this study did not find a statistical difference in perception of feedback between residents and attendings, additional factors should be considered when investigating perceived feedback deficiencies. Further research is needed to better understand and improve the clinical feedback process.

## Background

Feedback plays an important part in medical education and residency training. It has been studied for decades, and many authors have presented their own suggestions on how to deliver effective feedback with multiple definitions developed in the literature [1–4]. An early, widely-referenced paper by Dr. Jack Ende described feedback as “an informed, nonevaluative, objective appraisal of performance intended to improve clinical skills”, while a more recent literature review proposed a more detailed description, “a supportive conversation that clarifies the trainee’s awareness of their developing competencies, enhances their self-efficacy for making progress, challenges them to set objectives for improvement, and facilitates their development of strategies to enable that improvement to occur” [1, 5].

As the concept has proven difficult to define, it is not surprising that a consensus has not been reached for an ideal model for the delivery of feedback. Nonetheless, the importance of feedback remains unquestioned; as it can allow trainees to take a more critical evaluation of their performance, learn from previous mistakes and greatly improve their clinical skills in future practice. The American College of Graduate Medical Education (ACGME) has placed an emphasis on the importance of feedback by annually surveying residency programs and inquiring as to the depth of feedback that residents feel they receive within their program.

Various impediments to feedback are frequently cited. Lack of time for feedback delivery is often reported anecdotally, though direct evidence for this is difficult to find [3]. Though there are many styles and methods of providing feedback (e.g., written, formal in-person feedback sessions, and informal feedback occurring in real-time that can be considered [6]), feedback delivery can still be quite challenging for the person assigned the task of doing so. Some have suggested that the process may be inhibited due to fears of retaliation or a desire to preserve a good working relationship [7, 8]. However, recent evidence confirms that residents do want to receive feedback, particularly on higher-risk procedural skills [9].

One of the less-studied challenges related to clinical feedback involves the identification of what forms or aspects of feedback residents believe are effective. For example, faculty may provide feedback to residents during brief bedside encounters, yet, given the informal nature, this may not be fully appreciated as such by residents and other learners. Yarris and colleagues identified differences in resident versus attending perceptions of specific aspects of clinical feedback. Of note, the attendings in that study tended to report higher satisfaction with the quality of the feedback they gave than the residents did for the feedback they received. [10] Along the same lines, it has also been observed that medical students and their teachers disagree on the frequency of feedback received or given, respectively [11]. This suggests there may be a disconnect in teacher-learner perceptions of feedback within the field of medical education.

Van de Ridder et al. reviewed the literature and identified 33 factors that affected feedback, yet very little was found that addressed the feedback provider’s and recipient’s perceptions of the quality of that feedback [12]. Liberman et al. found significant differences in resident versus attending perceptions of the frequency and quality of feedback [13]. The reasons for these differences in perception are unclear. This study begins to address this gap in knowledge by characterizing what encounters faculty and residents identify as feedback and which forms are felt to be the most clinically effective by emergency medicine attendings and residents.

## Methods

Prior to initiating this study, ethics approval was sought from the local institutional review board (IRB). This study was deemed exempt from IRB approval, as it had no direct connection to patients or personal protected information and, therefore, did not represent human subjects research. No funding was utilized for this project.

An electronic survey was created using REDCap [14] and circulated via email to all emergency medicine and general surgery residents and attending physicians (both full-time and part-time) at Mayo Clinic in Rochester, MN. Attendings in both the adult and pediatric emergency departments were included, regardless of the type of residency completed, and all responses were anonymous. Residents rotating in the emergency department from other services (including pediatrics residents) and medical students were excluded from this study. This sample size was estimated a priori to be adequate to detect a 20% difference in perception between the two groups with a power of approximately 0.8. The survey was administered in September to allow new residents beginning in July time to gain experience in receiving feedback during residency before completing the survey.

Within the survey, subjects were presented with five clinical scenarios and asked to determine whether or not they believed it constituted effective feedback. Each scenario was followed by the question, “Which of the following is the best description of this interaction?”; and responses were limited to “this is effective feedback” or “this is not effective feedback”. These survey scenarios can be found in Appendix A. The scenarios were created by the authors as representations of typical resident/faculty interactions at their institution, and there was not an attempt to make an a priori determination of which scenarios did and did not represent effective feedback. Prior to starting data collection, the surveys were reviewed by a total of three attendings and five residents for clarity and to determine if they were representative of a typical interaction.

Continuous features were summarized with medians, interquartile ranges (IQRs), and ranges; categorical features were summarized with frequency counts and percentages. Comparisons of features between residents and attendings were evaluated using Wilcoxon rank sum, chi-square, and Fisher exact tests. Statistical analyses were performed using version 9.4 of the SAS software package (SAS Institute, Inc.; Cary, NC). All tests were two-sided and *p*-values < 0.05 were considered statistically significant.

## Results

Surveys were sent to 110 individuals. Of those, 72 (65.5%) responded to the survey, including 35 (49%) residents and 37 (51%) attendings. Of the 35 residents, 31 indicated their level of training, which included 13 (42%) PGY-1, 9 (29%) PGY-2, 6 (19%) PGY-3, 3 (10%) PGY-4, and none for PGY ≥ 5, respectively. Survey results include an equal breakdown of responses from residents in emergency medicine and general surgery. Attending physician responses are primarily representative from emergency medicine. Of the 37 attendings, 34 indicated the number of years since completion of residency or last fellowship, at a median of 9 years (IQR 4–14; range 1–31). One value for years since completion of training was set to missing, because an illogical value was entered by one respondent that likely represented the respondent’s age.

A comparison of the remaining survey responses between residents and attendings is shown in Table 1. Sample sizes for responses with missing data are indicated in italics in parentheses. In each of the five clinical scenarios there was not a statistically significant difference in what residents and attending physicians perceived as effective feedback. Each question of the survey demonstrated similar perceptions from residents and attending physicians with *p*-values ranging from 0.084 to 1.0.

**Table 1:** Results of Feedback Survey

	All *N* = 72	Residents *N* = 35	Attendings *N* = 37	
Feature	*Median (IQR; Range)*			*P*-value
Age in years (*N = 35;34*)	32 (30–42; 23–70)	30 (27–32; 23–37)	42 (35–47; 30–70)	< 0.001
Gender	*N (%)*			
Female	26 (36)	13 (37)	13 (35)	0.86
Male	46 (64)	22 (63)	24 (65)	
Department				
Emergency medicine	50 (69)	16 (46)	34 (92)	< 0.001
General surgery	22 (31)	19 (54)	3 (8)	
Question 1				
Effective feedback	65 (90)	30 (86)	35 (95)	0.25
Not effective feedback	7 (10)	5 (14)	2 (5)	
Question 2				
Effective feedback	67 (93)	33 (94)	34 (92)	1.0
Not effective feedback	5 (7)	2 (6)	3 (8)	
Question 3				
Effective feedback	27 (38)	14 (40)	13 (35)	0.67
Not effective feedback	45 (63)	21 (60)	24 (65)	
Question 4				
Effective feedback	68 (94)	33 (94)	35 (95)	1.0
Not effective feedback	4 (6)	4 (6)	2 (5)	
Question 5				
Effective feedback	20 (28)	13 (37)	7 (19)	0.084
Not effective feedback	52 (72)	22 (63)	30 (81)	
Overall Number Effective				
1	1 (1)	0	1 (3)	0.58
2	5 (7)	3 (9)	2 (5)	
3	34 (47)	16 (46)	18 (49)	
4	26 (36)	11 (31)	15 (41)	
5	6 (8)	5 (14)	1 (3)	

## Discussion

Feedback continues to play an important part in professional development. Yet, while the practice of medicine strives to be evidence-based in the twenty-first century, the practice of delivering feedback to medical trainees lags behind in this regard. The optimal format and delivery of feedback remains unknown, though recommendations can be found in the literature. Some have suggested that training is helpful to improve objective measures of clinical supervisors’ proficiency in providing feedback to residents. [15, 16]. Others have suggested that additional resources are needed to train clinicians to provide meaningful and formative feedback [7]. It has also been demonstrated that residents can provide valuable l feedback on the quality of feedback their educators provide. This suggests a role for bidirectional feedback [17, 18]. These interventions presume a shared perception of how feedback should be provided and received, but in many instances there may not be perception compatibility between teacher and pupil. However, in many instances this has yet to be clearly established.

This study investigated one of the foundational concepts of feedback; namely, do those giving and receiving feedback agree on what is feedback. In this limited, single-institution study, we have found evidence that they do agree on this aspect of feedback. This suggests that when feedback in medical training is thought to be suboptimal or ineffective, it may not be because of a fundamental disagreement between the involved parties disagree on what feedback is. Rather, consideration should be given to the delivery and content of the feedback.

This study was not designed to investigate these aspects of feedback, but a trend is suggested by the results. For three of the five scenarios, the responses for both the resident and attending groups overwhelmingly favored one response over the other. More than 85% of individuals thought effective feedback had been given in questions 1, 2, and 4. Opinions were split over the other two scenarios (scenarios 3 and 5) within both the resident and attending groups, though smaller majorities thought scenarios 3 and 5 did not include any effective feedback. The three scenarios that large majorities of both residents and attendings said did include effective feedback (scenarios 1, 2, and 4) were similar in that a specific recommendation was made for future clinical practice (e.g., “On your next needle decompression make sure to…”). Scenarios 3 and 5 did not include such a recommendation, but, instead, included only a more generalized statement that lacked actionable information (i.e., “try to place it faster” and “nice job today”. This suggests that both attendings and residents believe feedback should include actionable information, whether it is a specific recommendation for future improvement or recap of specific behaviors that were effective and warrant repeating, and is consistent with prior recommendations in the literature [1]. Future research may be useful to better identify what content is necessary for feedback to be interpreted as useful by residents and attendings.

This study is limited by the fact that participants were aware of the purpose of the study, and may have had a heightened perception of feedback that may not have been present in a real life encounter (e.g., Hawthorn effect). Additionally, the study utilized a relatively small sample size. Consequently, it was not possible to control for all variables that have been shown to affect feedback. It was, however, large enough to permit the detection of clinically meaningful differences in study results. The response rate differed by department of origin, with a much higher number of emergency medicine residents than general surgery residents responding. Further, all respondents worked at a single institution, thus limiting the diversity of the sample. There was not significant power to break results down by year of residency training. A prior study found that medical students perceptions of feedback changed as they progressed in their training [19]. It is possible that this may also be affected by level of residency training, although data on this are limited.

## Conclusions

This was a small, single-center study comparing resident and attending physician perception of feedback. Despite its size, there was fairly good agreement between the two groups as to which scenarios did and did not represent effective feedback. This suggests that resident and attending physicians tend to agree on what constitutes effective feedback. Thus, focus should be directed to other factors, such as feedback content, when there is a perceived deficit in quality feedback. Further research is necessary to identify an optimal model or models for delivery of effective clinical feedback.

## Appendix

### Study Feedback Scenarios

This contains the full text of the five example clinical feedback scenarios used in this study, along with the response choices presented to survey respondents.

1. A 65 year old male presents with respiratory distress. On physical exam, breath sounds are noted to be absent on the right and trachea is deviated to the left. The resident preforms a needle decompression on the right to relieve his tension pneumothorax. A 14 gauge angiocatheter is placed halfway between the 2nd and 3rd rib along the midclavicular line. A whoosh of air is heard and the patient does have a slight improvement in his work of breathing. The consultant* says, “Good job! Your patient’s symptoms have improved and he has temporarily stabilized. On your next needle decompression make sure to place your need just superior to the rib to avoid injuring the intercostal vessels and nerves.”

Which of the following is the best description of this interaction?

A) This is effective feedback
B) This is not effective feedback

2. The patient is now temporarily stabilized but needs a chest tube placed for management. The resident suggests using a 36 French thoracostomy tube. The consultant responds, “In a trauma patient that is a good option but in this case, use a pigtail catheter. It will be less uncomfortable and invasive for the patient and will achieve similar results.”

Which of the following is the best description of this interaction?

A) This is effective feedback
B) This is not effective feedback

3. A pigtail catheter is inserted and attached to the atrium with bubbling noted and significant improvement in the patient’s respiratory status. After CXR is obtained that demonstrates good positioning of the pigtail catheter and resolving PTX, the consultant asks, “How did you feel that went?” The resident responds, “It seemed to be tolerated well by the patient but I felt a little unsure if I was in the correct position.” The consultant answers, “Yes that is the difficult aspect of using a pigtail catheter. You were moving a little slow during the procedure. Next time try to place it faster.”

Which of the following is the best description of this interaction?

A) This is effective feedback
B) This is not effective feedback

4. Midway through the shift there is a lull in the number of patients and the consultant suggests you step into the conference week for feedback on how the shift is going so far. The consultant says, “You did a great job at quickly identifying the patient’s pneumothorax. Your clinical knowledge is very strong. The procedure may have gone smoother if your equipment was all laid out in order of use so that you know you have everything you need. Is there anything that I could be doing differently in the shift?”

A) This is effective feedback
B) This is not effective feedback

5. The end of a busy shift comes and the resident and consultant are leaving for the day. Prior to leaving, the consultant concludes with, “Nice job today!”

Which of the following is the best description of this interaction?

A) This is effective feedback
B) This is not effective feedback

*Term used at the study hospital for attending physician.

## Abbreviations

ACGME: Accreditation Council for Graduate Medical Education
IQR: Interquartile range
IRB: Institutional review board
PGY: Postgraduate year
